# A comparison of X-ray and calculated structures of the enzyme MTH1

**DOI:** 10.1007/s00894-016-3025-x

**Published:** 2016-06-27

**Authors:** Hannah Ryan, Megan Carter, Pål Stenmark, James J. P. Stewart, Sonja B. Braun-Sand

**Affiliations:** Department of Chemistry and Biochemistry, University of Colorado Colorado Springs, Colorado Springs, CO 80918 USA; Department of Biochemistry and Biophysics, Stockholm University, S-106 91 Stockholm, Sweden; Stewart Computational Chemistry, 15210 Paddington Circle, Colorado Springs, CO 80921 USA; In Silico Chemical Consulting, 128 Longwood Ave, Lakeway, TX 78734 USA

**Keywords:** Binding site, Enzyme, MTH1, Nudix box, 8-oxo-dGMP, PM7, Salt bridges

## Abstract

Modern computational chemistry methods provide a powerful tool for use in refining the geometry of proteins determined by X-ray crystallography. Specifically, computational methods can be used to correctly place hydrogen atoms unresolved by this experimental method and improve bond geometry accuracy. Using the semiempirical method PM7, the structure of the nucleotide-sanitizing enzyme MTH1, complete with hydrolyzed substrate 8-oxo-dGMP, was optimized and the resulting geometry compared with the original X-ray structure of MTH1. After determining hydrogen atom placement and the identification of ionized sites, the charge distribution in the binding site was explored. Where comparison was possible, all the theoretical predictions were in good agreement with experimental observations. However, when these were combined with additional predictions for which experimental observations were not available, the result was a new and alternative description of the substrate-binding site interaction. An estimate was made of the strengths and weaknesses of the PM7 method for modeling proteins on varying scales, ranging from overall structure to individual interatomic distances. An attempt to correct a known fault in PM7, the under-estimation of steric repulsion, is also described. This work sheds light on the specificity of the enzyme MTH1 toward the substrate 8-oxo-dGTP; information that would facilitate drug development involving MTH1.

**Graphical Abstract:**
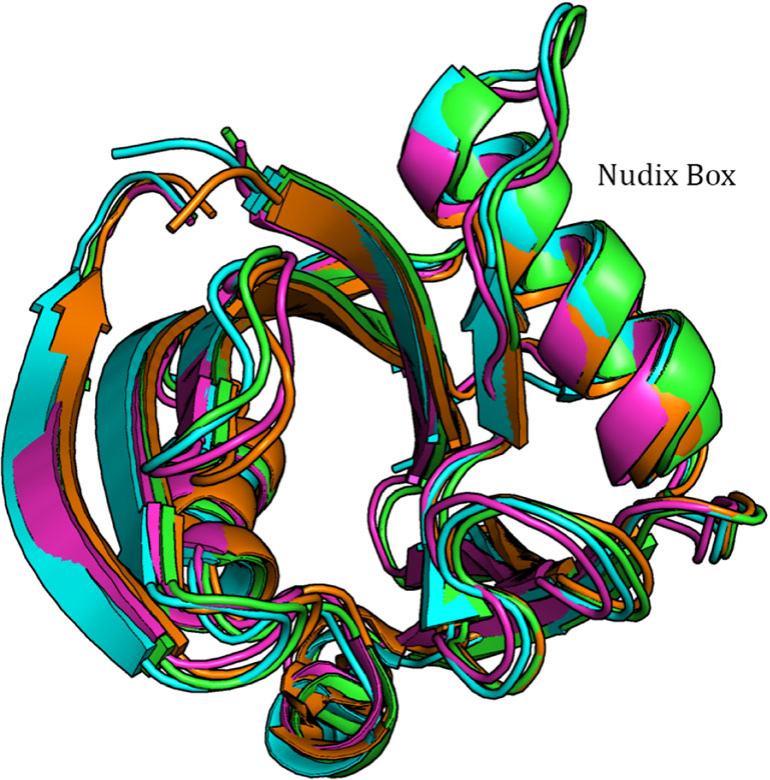
Overlay of the backbone traces of the two MTH1 protein chains (green and orange respectively) in PDB 3ZR0 and the equivalent PM7 structures (magenta and cyan respectively) each optimized separately.

Overlay of the backbone traces of the two MTH1 protein chains (green and orange respectively) in PDB 3ZR0 and the equivalent PM7 structures (magenta and cyan respectively) each optimized separately.

## Introduction

Protein structures have been obtained using X-ray analysis for many decades. Because of the tremendous importance of the results—access to accurate three-dimensional structures of large biological systems in the Protein Data Bank [1] (PDB)—the significance of this achievement is hard to overstate. In one field in particular, X-ray crystallographic results have provided information that could not otherwise have been available: specifically, structural information regarding the active sites and/or allosteric binding sites in enzymes. This is an essential prerequisite for investigating mechanisms in enzyme-catalyzed reactions. By combining structural information from the PDB and experimental results of enzyme behavior, many enzyme-catalyzed reaction mechanisms have already been worked out [2]. With the advent of modern computational chemistry methods the static picture of an X-ray structure can be used as the starting point for exploration and simulation of enzyme-catalyzed mechanisms, and thus promises to be an extremely powerful tool for modeling biochemical processes.

Weakly scattering atoms, such as hydrogen atoms, are not routinely discernible in X-ray determined structures. Because of this, all but the highest resolution X-ray structures in the PDB lack experimentally-determined hydrogen atoms. A second limitation toward exploring the catalytic mechanism of enzyme reactions is that, although X-ray analysis provides an accurate secondary and tertiary structure, the relative position of the constituent atoms is of limited accuracy. This accuracy is dependent in part on the resolution of the data collected. For a structure solved to 2.5 Å resolution, for example, one can expect atom location to vary by ± 0.4 Å compared to a structure solved to 0.8 Å, where one can expect atom locations to be accurate to 0.1 Å. This occurs for various reasons, some intrinsic to the nature of the crystal being analyzed and some as a result of technical issues, and illustrates a fundamental difference between X-ray analysis and biochemistry. Small discrepancies in interatomic distances, typically in the order of a fraction of an Ångstrom, are negligible from a physics perspective, but are of importance in determining biochemical reaction outcomes: the magnitude of the transition barrier and overall reaction energies involved in modeling enzyme-catalyzed mechanisms is likely to be small relative to the energetic cost of discrepancies in bond lengths [3]. Until both limitations—the small discrepancies in interatomic separation and the absence of hydrogen atoms—are solved, a significant number of catalytic mechanisms cannot be mapped solely using PDB coordinates.

One attempt [3] at overcoming these obstacles involves optimization of the structure derived from X-ray crystallography using the PM7 [4] semiempirical computational method, a method that has been shown to be effective in accurate hydrogen placement and predicting interatomic bond lengths, to generate new structures that, from a chemical perspective, are much more accurate than those obtained using either method on its own.

Various computational methods have been developed for modeling proteins. Where non-covalent interactions are important, molecular mechanics and molecular dynamics methods are preeminent. Such methods have useful accuracy and speed, but, because they are purely mechanical, they are not suitable for modeling reactions. For modeling reactions, *ab initio* quantum mechanics (QM) techniques, such as Hartree-Fock and density functional theory methods, dominate. These methods are the most accurate, but because they are computationally very demanding, in order to be useful in modeling enzymes they are normally only used for modeling the active site, the rest of the protein being modeled using molecular mechanics (MM) methods. In turn, this combination of two very different techniques, the QM/MM method [5–9], introduces new problems, particularly at the interface between the QM and MM regions, but for many applications the resulting benefits of improved accuracy and reduced computational effort fully justify the additional effort involved in setting up the simulation.

An alternative to the QM/MM approach is to use semiempirical methods. These are two to three orders of magnitude faster than *ab initio* methods when applied to large systems, and, when used with a linear scaling technique, such as MOZYME [10], an even greater speed can be achieved. The resulting methods permit geometric operations to be routinely performed on systems of several thousand atoms —in this study, over 5,000 atoms—in a day using conventional desktop computers, or, in the case of the initial geometry optimization, a few days.

Several semiempirical methods, PM6 [11], PM6-DH2 [12], PM6-DH+ [13], PM6-D3H4 [14], and PM7 [4] are suitable for modeling proteins. All these methods are currently available in the program MOPAC2012 [15] and its successor, MOPAC 2016 [16]. Of these methods, the recently-developed PM7 was selected based on its overall performance [4]. During the development of PM7, a survey was made [4] of the ability of PM7 to reproduce the overall structure of proteins. Subsequent work confirmed the accuracy of the PM7 method [3] and resulted in the demonstration of how PDB structures could be improved by using PM7. More recently, a fault was found [17] in PM7 where some pairs of protein residues that had only very weak non-covalent interactions were predicted to be unrealistically close together.

Using a single method to model an entire protein eliminates the problems encountered by using two methods, but there could still be issues caused by faults in the method. One such fault is the unexpected close contacts just mentioned, the presence of which could compromise the validity of work done in modeling properties of interest such as binding and reaction mechanisms. The objective of this investigation is to determine the strengths and weaknesses of using the PM7 method in this context.

Because of interest as a potential target for cancer therapeutics, human mutT homologue protein 1 (MTH1) was chosen to illustrate the relationship of the computed model and the crystal structure resulting from X-ray analysis. MTH1 selectively hydrolyses 8-oxo-2′-deoxyguanosine-5′-triphosphate, 8-oxo-dGTP, to the monophosphate, 8-oxo-dGMP. 8-Oxo-dGTP has been implicated in causing damage to DNA, and can therefore be regarded as mutagenic. By removing a pyrophosphate group from 8-oxo-dGTP, MTH1 effectively renders it inert, and thus makes it unavailable for incorporation into DNA. MTH1 is upregulated in cancerous cells, making the cells less susceptible to oxidative damage. MTH1 thus enables the rapid cell divisions necessary for the cancer to grow [18]. MTH1 has therefore recently gained interest as a target in anti-cancer research, and as such was chosen as a realistic and timely test system for this work [18].

**Fig. 1:**
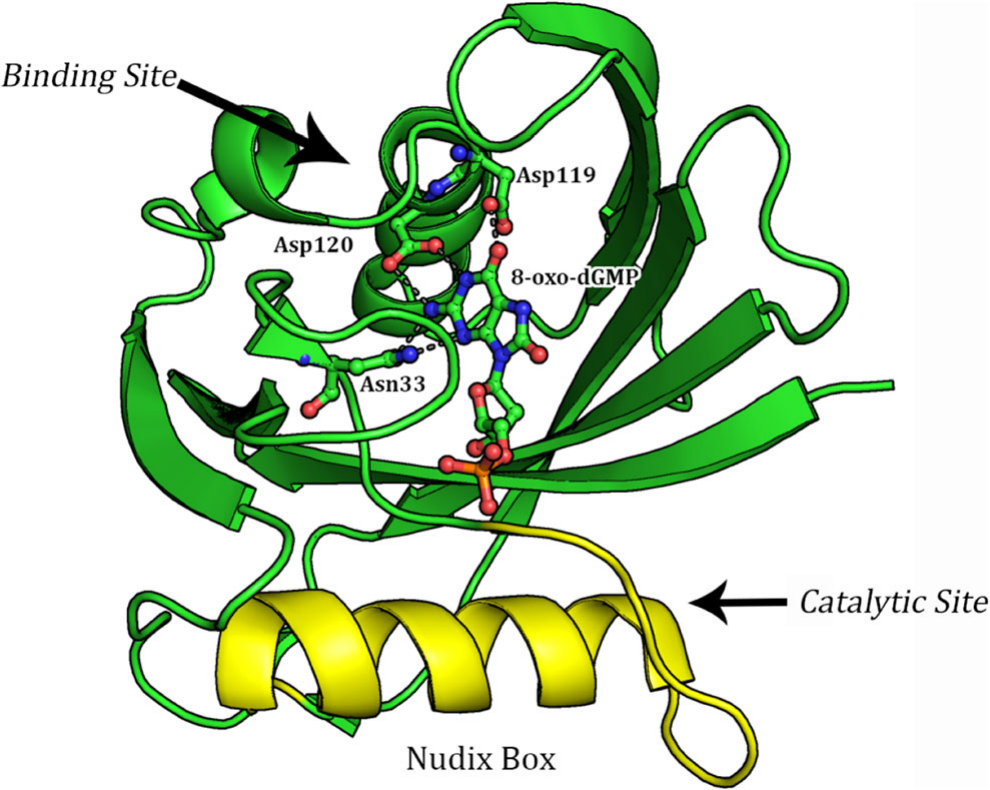
Cartoon structure of MTH1 (3ZR0) showing location and hydrogen binding interactions (*gray dash lines*) to the 8-oxo-dGMP product (stick model) relative to the catalytic Nudix box (*yellow*)

Unlike most enzymes in which the active site consists of a binding pocket where the catalytic reaction occurs, the binding and reaction sites in MTH1 are separated by several Ångstroms. This means that the binding site can be examined while ignoring the mechanism of hydrolysis, and the hydrolysis mechanism can be explored without regard for any binding considerations. In this study the environments of both the binding and catalytic sites will be examined (Fig. 1).

**Fig. 2:**
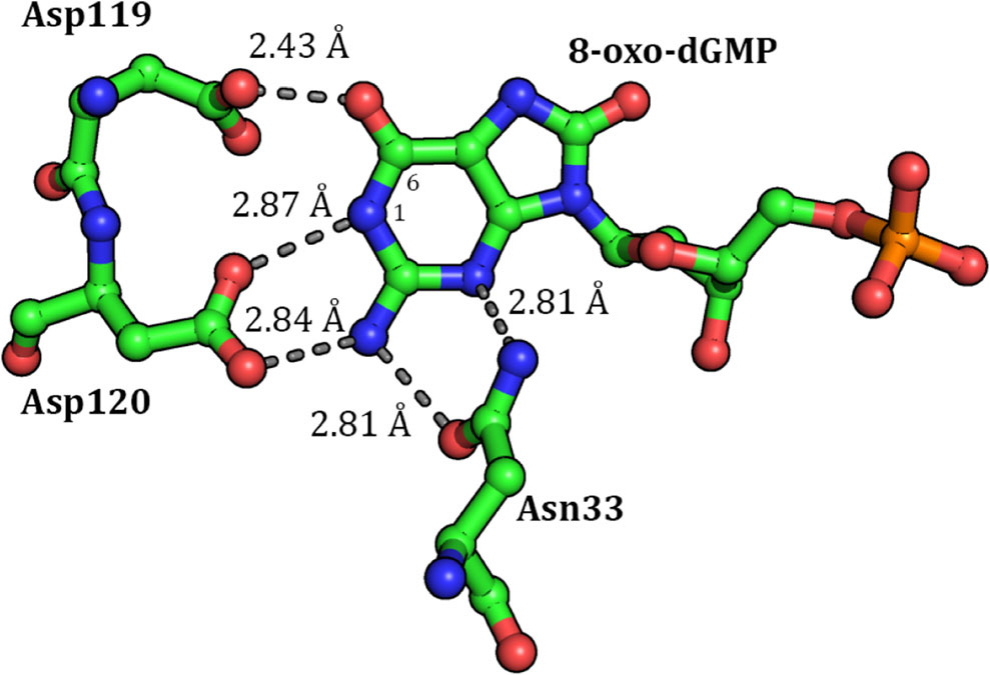
Binding site of 8-oxo-dGMP in 3ZR0, chain A, showing important hydrogen bond distances between the guanine group and the residues Asn33, Asp119, and Asp120

The environment of the binding site was explored in a recent [19] analysis of the crystal structure, PDB entry 3ZR0, of the MTH1 enzyme complexed with its product, 8-oxo-dGMP. Like most protein structures determined by X-ray analysis, hydrogen atoms were not located, so some electronic phenomena, such as the state of ionization of various sites and some intermolecular interactions, had to be inferred from the positions of the heavy atoms. The orientation of important residues in substrate binding and recognition are shown in Fig. 2. The binding site contacts consist of three MTH1 residues (N33, D119, and D120) and from the heavy-atom geometry these were inferred to form five hydrogen bonds with the 8-oxo-guanine group.

MTH1 has been shown to exhibit a preference for hydrolyzing 8-oxo-dGTP over dGTP, although the reasons for this are unclear. Most surprisingly, no direct interactions between MTH1 and the 8-oxo group of 8-oxo-dGMP were observed in the crystallographic structure 3ZR0. However, an examination of the structure of 3ZR0 has resulted in an alternative hypothesis. One of the putative hydrogen bonds, between one of the O_δ_ on D119 and O_6_ of 8-oxo-dGMP, is very short, the O-O distance being only 2.43 Å. Such a close hydrogen bonding contact (below 2.5 Å) is rare in proteins [20], but does occur when a carboxylate group is involved [21], and indicates the presence of a negative charge at this site. The authors’ initial hypothesis therefore was that the carboxylate group of D119 was negatively charged, forming a hydrogen bond with the enol tautomer of 8-oxo-dGTP. In their words the authors speculated [19] that “… the 1- and 6-position is critical for the specificity and that the 6-enol-8-keto form of 8-oxo-dGTP is able to donate and accept hydrogen bonds at these specific positions. The 8-oxo group of 8-oxo-dGTP makes the 6-enol form a more prominent and stable tautomer than for the un-oxidized nucleotide and suggests that significant levels of the 6-enol-8-keto form exists in solution, “and, that”… the basis for the difference in affinity between oxidized and un-oxidized nucleotide is that the 8-oxo modification of the base influences the keto-enol tautomerization and electrostatic properties of the opposite side of the base, which makes several key interactions with MTH1.” Unfortunately, because of the absence of hydrogen atoms, this hypothesis could neither be supported nor refuted by examination of the crystallographic structure.

The asymmetric unit of PDB entry 3ZR0 contains two different conformations of MTH1 proteins, each containing one molecule of 8-oxo-dGMP bound in the active site. In addition, there are five sulfate ions and 201 water oxygen atoms, for a total of 2750 atoms, before the addition of the hydrogen atoms. Each of the protein molecules and its associated small molecules and ions were defined by the chain letters A or B. Of the two macromolecules, MTH1 protein A was the better defined and consisted of a single chain of 154 residues, starting with residue 3. The other, MTH1 protein B, was less well defined, and consisted of two chains, one with residues 2-13 and one with residues 18-156 for a total of 151 residues. Both proteins were roughly spherical and exhibited the canonical NUDIX fold with an α-helix, β-sheet, and α-helix arrangement, where the helices reside on opposing sides of a mixed β-sheet. Two regions of interest in the tertiary structure are the catalytic site where hydrolysis occurs and the binding site for 8-oxo-dGMP.

### Methods

Ideally, a computational model should consist of two components: a theoretical method for simulating the phenomena in the system (binding, reaction, etc.), and a description of the chemical system itself (geometry). Obviously, if either component has a significant defect then no confidence can be placed in any results obtained by the simulation. To address this issue, and before proceeding further, it is essential to determine the validity of both components.

## Theoretical method

The semiempirical method PM7 [4] was used to computationally model MTH1. This method, as with all similar semiempirical methods, replaces the exact quantum-chemistry description of a system with a set of approximations, each of which consists of an algebraic expression and a set of adjustable parameters. These parameters are then optimized to give the best fit to reference data, typically properties such as heats of formation, geometries, dipole moments, and ionization potentials, for a training set of molecules and ions. These data are obtained from experiment and from high-level calculations. By using simple approximations, the resulting method is very fast compared with more sophisticated methods, and, by using reference data to define the adjustable parameters, a method can be developed that has useful accuracy.

In addition to a quantum mechanical self-consistent field (SCF) procedure, semiempirical methods such as PM7 are augmented by a small number of post-SCF modifications which are designed to improve the representation of intermolecular interactions. Of these, the most important are the addition of a dispersion correction [22] and a term to represent the hydrogen bond [4].

Using PM7 allows a single method to be used for modeling the whole protein. This obviates the need for specific methods for treating individual chemical structures, such as salt bridges and hydrogen bonds, thus avoiding the possibility of introducing specific errors into the energy and structural properties in those moieties.

An important consideration, particularly in biochemical systems, is the effect of the aqueous environment on the system being studied. One option for simulating the aqueous phase would be to surround the system being examined by a shell of water molecules, but this approach has two severe drawbacks. First, the computational effort would increase considerably, and, second and more importantly, the process of geometry optimization mimics the effect of minimizing the energy of the system. That is, geometry optimization simulates the effect of cooling the system to 0 K. Semiempirical methods are parameterized to reproduce properties, e.g., bond lengths and angles, etc., at 298 K, but simple, unconstrained geometry optimization generates a stationary point on the potential energy surface; such a point represents a system with no kinetic energy, i.e., a system at 0 K. To mimic in vivo temperatures a molecular dynamics simulation would be necessary. If explicit water molecules were used, the overall effect of optimizing the geometry would be to generate a model of a biochemical system surrounded by a shell of ice. Many methods for handling explicit water exist that avoid this result; these mimic liquid water by using dynamics simulations. Such simulations are slow when quantum chemical methods, even semiempirical methods, are used, and, if explicit water were used in such a simulation, the computational requirement would be prohibitive. An alternative, used here, is to simulate an implicit solvent using the COSMO technique [23], a dielectric continuum solvation model for determining the electrostatic interaction between a chemical system and a polarizable solvent, in this work, water. This is a fast and reliable method for including the aqueous medium in the computational model, and was used in all calculations reported here. The PM7 method has been validated extensively, and the results [4] indicate that it can reproduce the various types of structures and phenomena found in proteins with useful accuracy.

Systems involving enzymes are, of their nature, very large. Solving the SCF equations using conventional matrix algebra scales as the cube of the size of the system. As a result, these methods are impractical for routine use on biochemical macromolecules. An alternative method, MOZYME, based on localized molecular orbitals, scales almost linearly. MOZYME starts by constructing the Lewis structure for the system. This consists of highly localized M.O.s (LMOs), then, during the process of solving the SCF equations, the LMOs delocalize onto the surrounding atoms. When the SCF is achieved, the results for observables, in particular heats of formation and geometries, are identical, within arithmetic tolerance limits, to those obtained using conventional matrix algebra. Currently, the MOZYME method is limited to closed-shell systems, i.e., radicals and excited states cannot yet be handled, but, as all the systems involved in this work were closed shell, they represent ideal candidates for modeling using MOZYME.

### Construction of the model

Three starting systems were constructed from 3ZR0. The largest, referred to here as MTH1 A+B, consisted of the entire 3ZR0 system; the other two systems consisted of all atoms with chain label A, referred to as MTH1 A, and all atoms with chain label B, referred to as MTH1 B. Each of the smaller systems thus consisted of one molecule of MTH1, one molecule of 8-oxo-dGMP, associated sulfate ions, and water molecules. The composition of these systems plus added hydrogen atoms is shown in Table 1.

**Table 1:** Composition of the systems used

Species	Formula	MTH1 A+B			MTH1 A			MTH1 B		
		No.	No. Atoms	Net Charge	No.	No. Atoms	Net Charge	No.	No. Atoms	Net Charge
MTH1	A: C_807_H_1195_N_209_O_232_S_6_ B: C_788_H_1196_N_201_O_227_S_6_	2	4898	−8	1	2480	−4	1	2418	−4
8OG	C_10_ H_12_N_5_O_8_P	2	72	−2	1	36	−1	1	36	−1
Water	H_2_O	201	603	0	134	402	0	67	201	0
Sulfate	[SO_4_]^2−^	5	25	−10	2	10	−4	3	15	−6
Total			5598	−20		2928	−9		2670	−11

*MTH1*: Human mutT homologue protein

*8OG*: 8-Oxo-2′-deoxyguanosine-5′-monophosphate

### Hydrogenation

Most PDB files do not include hydrogen atoms, but, because a prerequisite for computational quantum chemistry modeling is that the chemical system should be complete in the sense that all valencies should be satisfied, they need to be added. Methods such as MolProbity [24, 25] and WHAT IF [26] can add hydrogen atoms to PDB structures. WHAT IF does a particularly good job of positioning hydrogen atoms in water molecules so that hydrogen bonds are optimized. MOPAC can also add hydrogen atoms and has options for selectively ionizing some or all of the ionizable sites. Hydrogen atom minimizations available in MOPAC also allow optimizing positions of hydrogen bonds. This flexibility made the use of MOPAC the preferred choice for this project. Hydrogenation was performed in two steps, the first of which consisted of adding hydrogen atoms to satisfy valence requirements. All sites ionized were readily-ionizable at physiological pH: these included Asp, Glu, Arg, and Lys residues, chain termini, sulfate (dianion), and the phosphate (monoanion) of 8-oxo-dGMP. For MTH1 A+B this amounted to 43 cationic and 63 anionic sites, resulting in a net charge of −20. This large net negative charge must be counterbalanced to satisfy the solid-state requirement of crystal formation and in part explains the low pH conditions required for crystal formation [19].

Hydrogenation was followed by optimizing the position of all hydrogen atoms while holding the positions of all other atoms fixed. Geometry optimization of the hydrogen atoms was carried out using PM7 and the L-BFGS [27, 28] optimizer in MOPAC2012. Optimization was considered complete when the calculated gradient norm dropped below 2.0 kcal∙mol^−1^∙Ångstrom^−1^, a value that gave the best balance between minimizing computational effort and obtaining an acceptable precision in the calculated ΔH_f_. Each structure in which the positions of the hydrogen atoms were optimized, while the positions of all other atoms were held fixed at the PDB coordinates, will be referred to as a “PDB starting geometry.” Optimization of the positions of hydrogen atoms in MTH1 A+B resulted in the formation of a large number of hydrogen bonds; an examination of PDB starting geometry for MTH1 A+B using JSmol [29] showed that, in all, 581 hydrogen bonds had formed. Using MOPAC and keyword “DISP” (to print dispersion and hydrogen bond information) confirmed that there were indeed 581 hydrogen bonds that had stabilizing energies greater than 1.0 kcal∙mol^−1^.

### Geometry optimization

Unconstrained geometry optimization of the three systems was then performed. Initially, an attempt was made to reduce the forces acting on the atoms, i.e., the gradient norm, to 2.0 kcal∙mol^−1^∙Ångstrom^−1^, but before this criterion was satisfied the calculated ΔH_f_ reached a minimum and started to fluctuate over a range of about two kcal∙mol^−1^. At this point, it was apparent that achieving the target gradient norm was impractical, therefore the termination criterion was changed so that the geometry was considered optimized if no decrease in energy occurred over a large number of optimization cycles (typically between 50 and 100 cycles). When this new condition was satisfied, the geometry that had the lowest ΔH_f_ was selected. At that point the gradient norm was on the order of 7–10 kcal∙mol^−1^∙Å^−1^, or about 0.1 kcal∙atom^−1^∙Å^−1^. The resulting geometry was then considered to be at a stationary point on the PM7 potential energy surface.

## Results

### Heat of formation

One measure of the difference in the PDB and PM7 geometries is provided by the change in ΔH_f_ when the PDB geometry is optimized using PM7. The difference in ΔH_f_ of the PDB starting geometry, -43073.6 kcal∙mol^−1^, and that of the PM7 optimized geometry, −46930.1 kcal∙mol^−1^, for MTH1 A+B was -3856.5 kcal∙mol^−1^. This change in ΔH_f_ could be attributed to two causes. Of its nature, the PDB X-ray geometry has small errors in the prediction of atom positions, typically on the order of a small fraction of an Ångstrom. These errors can result in changes of covalent bond lengths upon optimization. For example, one of the more extreme outliers in the PDB geometry occurs in Q54 in chain B, where the C_β_-C_γ_ covalent single bond length was reported to be 1.73 Å instead of the expected 1.53 Å. All distortions of this type result in energy penalties that result in an increase in the calculated ΔH_f_ of the starting geometry.

Semiempirical methods can reproduce covalent bond lengths of the type found in proteins within an accuracy of about 0.02 Å, and, when a PDB geometry is optimized, all errors of the type just described are corrected during the first few optimization cycles. However, current semiempirical methods, including PM7, have limited accuracy in the prediction of other geometric quantities such as interatomic distances between non-covalently bound atoms.

**Fig. 3:**
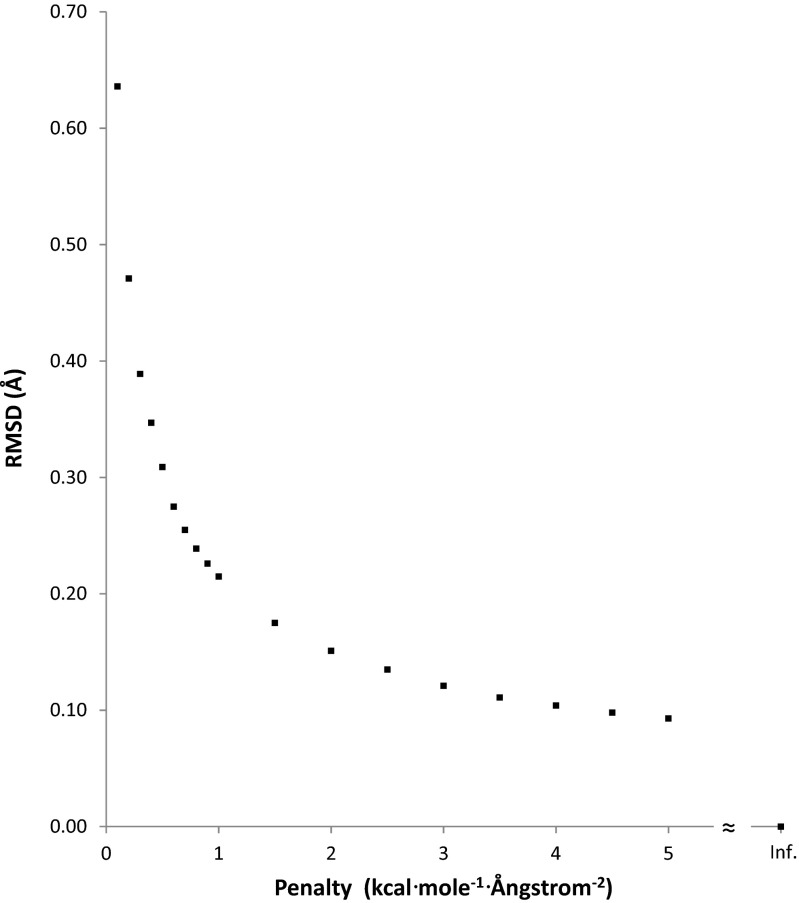
Variation in the root mean square difference (RMSD) between the PDB and PM7 geometry for MTH1 A+B as a result of different penalties toward the PDB structure

**Fig. 4:**
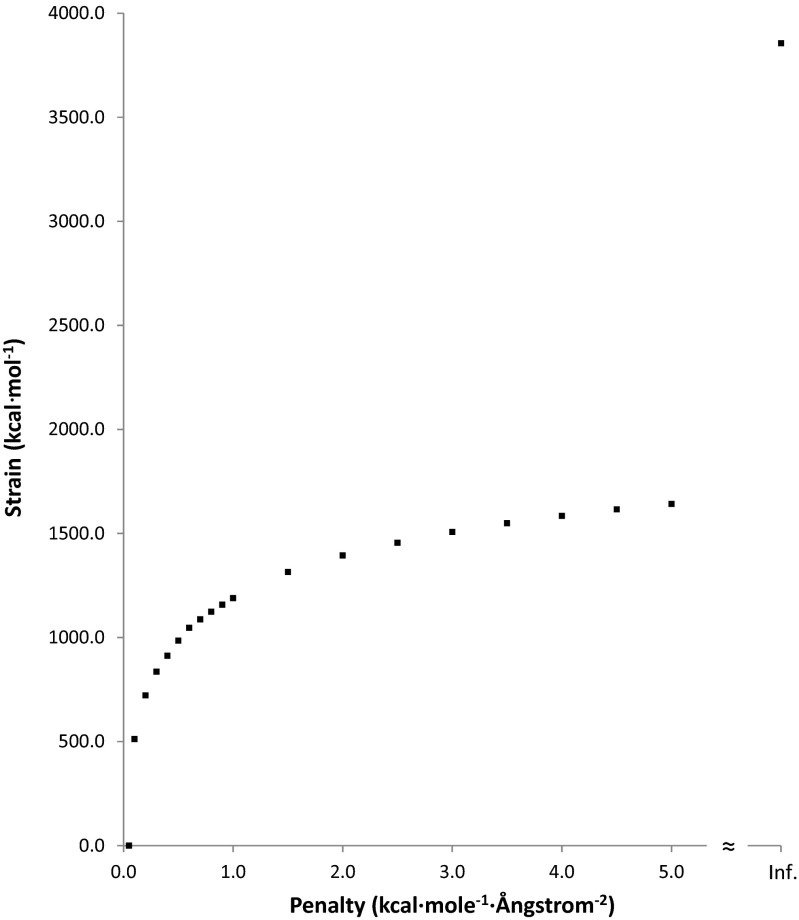
Variation in ΔH_f_ difference between PDB and PM7 geometry for MTH1 A+B as a result of different biases toward the PDB structure

Errors in PM7 have an associated energy contribution that decreases the ΔH_f_ of the PM7 optimized geometry relative to that of the hypothetical exact geometry, thus increasing the difference between the reference (3ZR0) and the optimized geometry. To investigate the contribution to the drop in ΔH_f_ attributable to faults in the PDB geometry and faults in the PM7 geometry, geometry optimizations were performed in which a set of different penalties were imposed. These penalties took the form of a potential [30] for deviation from the original PDB starting geometry. For MTH1 A+B, the effect of these penalties on the root mean square difference (RMSD) and on the drop in ΔH_f_ are presented in Figs 3 and 4, respectively.

### Additivity of fragments

**Fig. 5:**
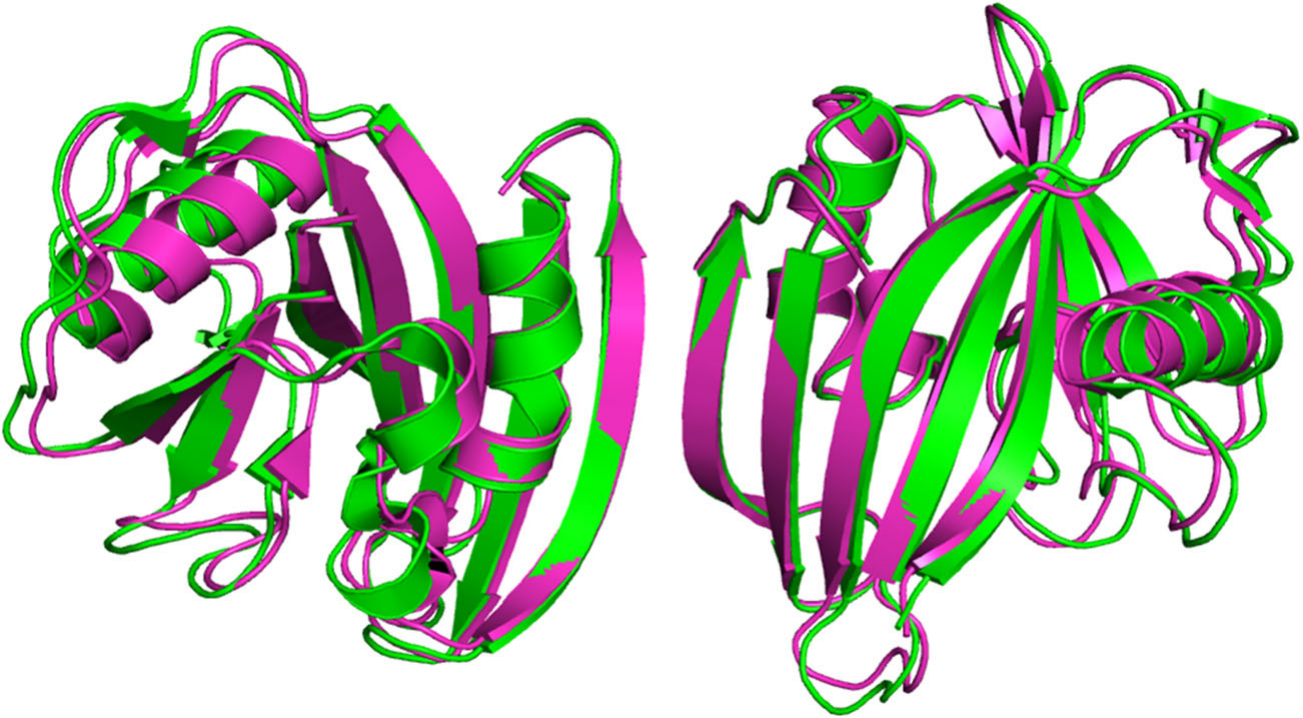
Comparison of MTH1 PDB entry 3zr0, in green, and PM7 optimized, in magenta, backbone structures

The computational efficiency of PM7 allows the entire crystallographic structure in 3ZR0 to be optimized. A comparison of the trace of the backbone structure of the PDB and the unconstrained fully optimized PM7 geometries for MTH1 A+B is shown in Fig. 5. Quantitatively, the PDB and PM7 geometries can be related by calculating the RMSD between them; for the fully optimized PM7 structure in which all ionizable sites were ionized, and the PDB starting geometry of 3ZR0, the RMSD (all atoms) was 1.37 Å. For MTH1 A plus associated moieties on its own, the RMSD was 1.15 Å, and for MTH1 B on its own, 1.21 Å.

**Fig. 6:**
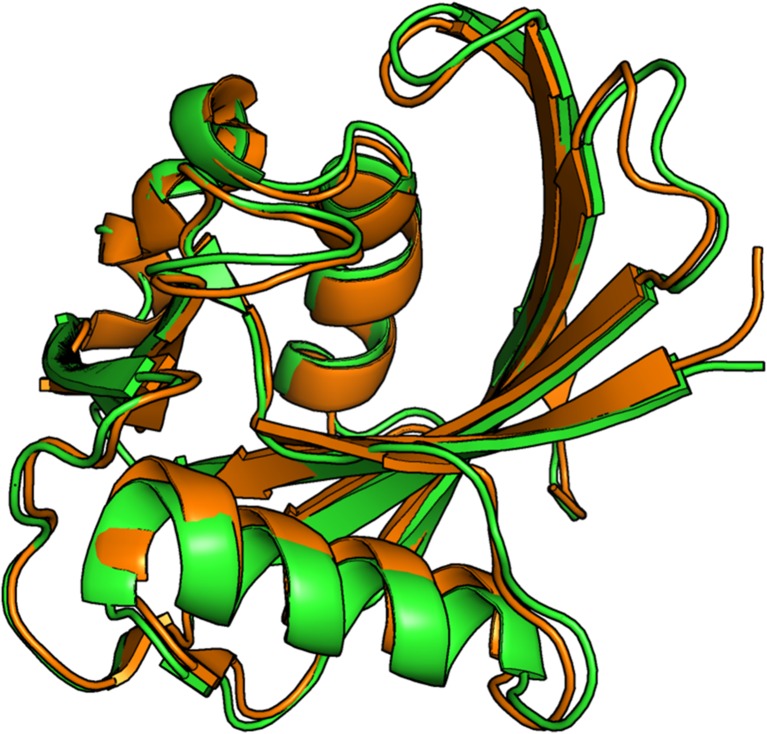
Superimposition of chain A (*green*) and chain B (*orange*) backbones in 3ZR0. The RMSD was 0.72 Å; for backbone atoms only: 0.38 Å

However, in order to minimize computational efforts when investigating individual molecular interactions (ionization, salt bridge formation, etc.) the size of the starting system was further reduced. Given that 3ZR0 is composed of two independent MTH1 proteins plus associated small molecules and ions, one way to reduce the computational effort in modeling the protein would be to use only one fragment. A second advantage of using this option would be that a more realistic description of the in vivo environment would be provided by a system that contained only one molecule of MTH1 instead of the dimeric system found in 3ZR0. A direct comparison of the 3ZR0 geometries of MTH1 A and MTH1 B (each containing 1218 atoms and consisting of residues 3-13 and 18–156) produced a RMSD minimum for the two molecules of 0.72 Å (Fig. 6). Given that the two MTH1 molecules are the same, most of this difference can be attributed to crystal packing. The RMSD of the 600 atoms of the backbone was 0.38 Å and that of the side chains (618 atoms not in the backbone) was 0.93 Å demonstrating that, as expected, most of difference observed involved movement of the side-chains.

**Fig. 7:**
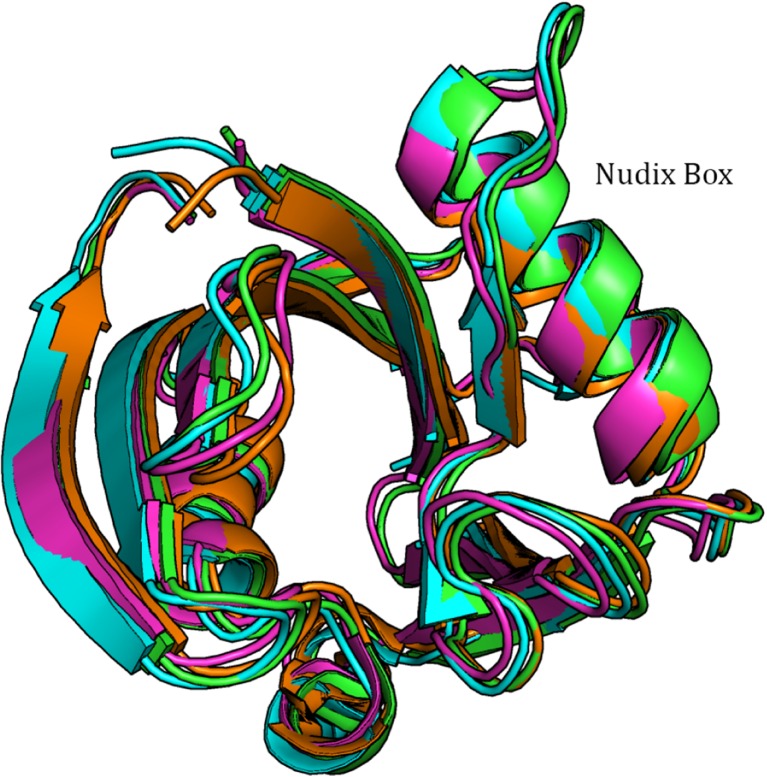
Overlay of the backbone traces of the two MTH1 protein chains (*green and orange* respectively) in PDB 3ZR0 and the equivalent PM7 structures (magenta and cyan respectively) each optimized separately

To test the validity of using only one chain in modeling MTH1 during optimization, the geometry and energetics of modeling MTH1 A+B were compared to those for MTH1 A plus MTH1 B; each being modeled separately. The geometry resulting from optimizing only one molecule of MTH1 plus associated systems was similar to that of the same system when the entire 3ZR0 was used. All atoms in the fully-optimized MTH1 A and MTH1 B systems were overlaid with each other and the assembly then overlaid with the equivalent atoms in the X-ray structure of MTH1 A and MTH1 B, as shown in Fig. 7. A comparison of the various structures shows that the differences between the PM7 and PDB structures is similar in magnitude to the differences between the two PDB structures.

Another measure of additivity would be to compare the ΔH_f_ of fully-optimized MTH1 A+B (-46930.1 kcal∙mol^−1^) with those of MTH1 A (−25909.6 kcal∙mol^−1^) and MTH1 B (−20862.1 kcal∙mol^−1^); this showed that MTH1 A+B was more stable by 158.4 kcal∙mol^−1^. A perfect fit could not be expected, in that, when 3ZR0 was split into two fragments, there would be a change in energy due to the loss of intermolecular interactions, which consist mainly of 16 hydrogen bonds, and the concomitant exposure of the new surface to implicit solvation. This energy increase, resulting from the loss of hydrogen bonds, would then be partially offset by the drop in energy arising from the increased surface area exposed to solvation. That is, there would be a partial cancellation of energy terms, resulting in the small change in ΔH_f_ observed.

These results indicate that there were no discernible phenomena specifically associated with using the entire 3ZR0 system, so no further work was done on this system and instead only systems containing a single MTH1 protein were examined.

### Prediction of charged sites

Strong experimental evidence exists that proteins in vivo contain charged sites and that often these charges are not balanced, i.e., that the protein has a net charge. To verify that the computational model being used would be able to predict ionization sites in MTH1, the effect of changing the ionization state of various sites was investigated.

A system consisting of the heavy atoms in MTH1 B was hydrogenated so that all ionizable sites were neutralized except for D119 because of the short O_δ_-O_6_ distance observed in 3ZR0, and the positions of all hydrogen atoms were then optimized. This system was artificial, in that readily-ionized moieties such as H_2_SO_4_ would naturally exist in an ionized state, but, for this study, almost complete hydrogenation was chosen in order to explore salt bridges. This system had a calculated ΔH_f_ of -17837.8 kcal∙mol^−1^. Examination revealed 16 potential salt bridges. Each of these salt bridges was then constructed by moving a proton from one residue to the other, and the positions of all hydrogen atoms re-optimized. In every case, the ΔH_f_ decreased, as shown in Table 2. An estimate of the interaction energy between salt bridges was obtained by calculating the energy of the system when all 16 salt bridges were present; this gave a value of -18122.2 kcal∙mol^−1^. Starting with the ΔH_f_ of the uncharged system and adding in the individual stabilization energies for the 16 salt bridges calculated individually (-293.6 kcal∙mol^−1^) yielded a predicted ΔH_f_, assuming no salt-bridge to salt-bridge interactions, of -18131.4 kcal∙mol^−1^. The difference between the two ΔH_f_, 9.2 kcal∙mol^−1^, or 0.6 kcal∙mol^−1^ per salt bridge, could then be attributed to interactions between salt bridges and to numerical instability in the calculations.

**Table 2:** Salt bridges and ionized sites in MTH1 B complexed with 8OG used in determining additivity of salt-bridge energies

Cation	Anion	Dist.^1^	ΔH_f_	Diff.^2^
Arg151	HSO_4_ 1159	2.46	−17883.3	−45.5
Lys23	8OG 1157	2.61	−17840.9	−3.1
Lys24	Glu97	2.64	−17857.1	−19.3
Arg50	Glu46	2.70	−17865.6	−27.8
Lys131	Glu152	2.75	−17858.6	−20.8
Arg51	Glu43	2.82	−17850.2	−12.4
Arg31	Asp143	3.18	−17853.2	−15.5
Lys114	Asp115	3.26	−17854.2	−16.4
His84	Asp82	3.51	−17862.1	−24.3
Lys66	Val156	3.96	−17841.0	−3.3
Arg5	Glu79	4.11	−17858.0	−20.2
Lys138	Glu73	4.54	−17855.0	−17.2
Lys138	Asp147	4.94	−17845.5	−7.8
His134	HSO_4_ 1158	5.46	−17877.3	−39.5
Lys132	Val156	6.08	−17844.7	−6.9
Lys130	Asp89	6.75	−17851.4	−13.6

1: Smallest nitrogen-oxygen distance, in Å

2: Difference in ΔH_f_ between un-ionized system, ΔH_f_ = −17837.8 kcal∙mol^−1^, and system with salt bridge, in kcal∙mol^−1^

**Fig. 8:**
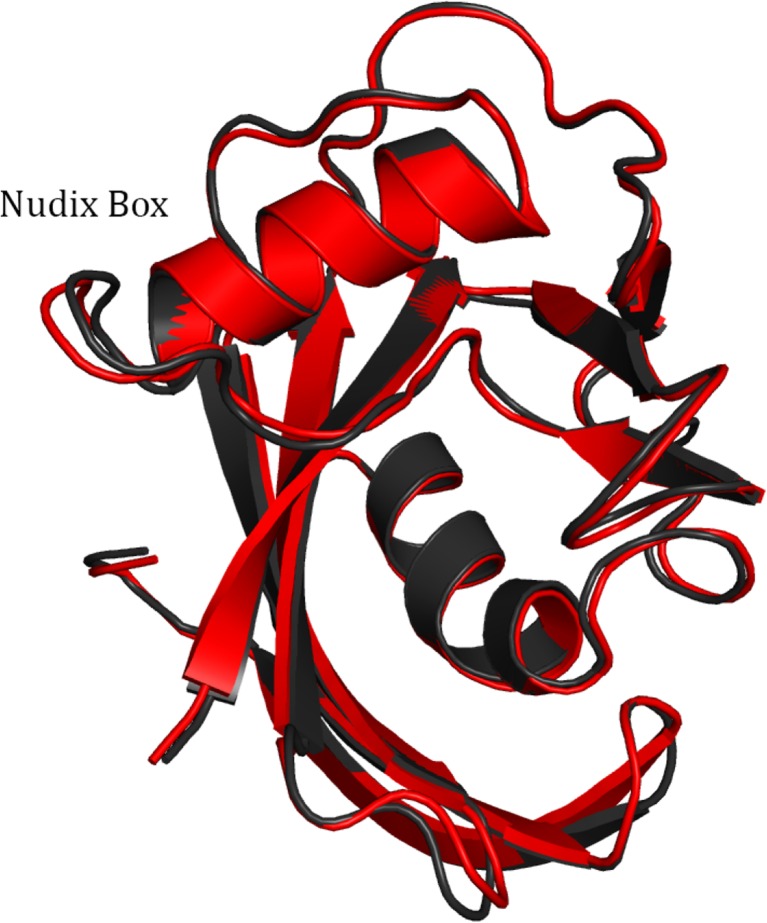
Trace of backbone of fully optimized MTH1 + 8-oxo-dGMP for neutral (*black*) and completely ionized (*red*) systems

Identification of all the sites in a protein that are ionized can be difficult. Some, such as those involved in salt bridges, are straightforward, while some can be inferred from an examination of their environment, and some might be difficult to predict *a priori*. Of interest, then, is determining the importance of correctly identifying the charged sites and the geometric consequences resulting from ionized sites not being correctly identified and defined in the system being modeled. A comparison was made of the fully-optimized geometries of chain A, in which all ionizable sites were ionized, and the equivalent system in which all sites except one were neutral at the start of the optimization. When the two geometries were superimposed the resulting RMSD was 0.77 Å. For the backbone atoms on their own (Fig. 8), the RMSD was significantly smaller, 0.49 Å, implying that motion of the side-chains was the main contributor to the RMSD.

The small change in the backbone geometry in going from all-ionized and all-neutral residues implies that the ionization state of most residues is unimportant when sites of interest are being investigated, the exception being those residues in, or near to, the site of interest. Strong hydrogen bonding networks exists at both sites of interest in MTH1. These provide additional rigidity so that the effect of changing the ionization state of a residue far from these sites would be reduced even further.

### Spontaneous salt bridge formation

The 18 geometries (comprising one in which all sites were un-ionized, 16 with individual salt bridges, and one with all but one site ionized) resulting from hydrogen atom optimization were then used in unconstrained geometry optimizations. As expected, there was a dramatic decrease in the ΔH_f_ of over 2000 kcal∙mol^−1^, but, when the various heats of formation were compared, the expected additivity of salt bridge stabilization energies was not present; indeed, two of the individual salt-bridge systems had heats of formation similar to that of the un-ionized system. Examination of these optimized structures of MTH1 B molecule showed that all three systems contained the same three salt bridges, R51-E43, K131-E152, and K23-E56, which had formed spontaneously during the geometry optimization, a phenomenon that had not occurred in the previous set of optimizations in which the heavy atoms had been kept fixed. An examination of the other single salt bridge systems showed that extra salt bridges had formed spontaneously in every one during the geometry optimization. These results are definitive evidence that the computational model being used was able to predict the existence of charged sites in MTH1.

When an equivalent unconstrained all-atom geometry optimization of MTH1 A was performed, starting with a completely neutral system, only the K23-E56 salt bridge formed spontaneously. Inspection of the K131-E152 region showed that the smallest oxygen-nitrogen distance was 5.70 Å, an interatomic separation so large that a spontaneous, i.e., activationless, migration of a proton from the carboxylic acid to the amine group would be unlikely. In MTH1 B, the equivalent distance was 2.75 Å.

In MTH1 A, the third potential salt bridge, R51-E43, was ideally positioned for a proton migration from E43 to R51, with both oxygen-nitrogen distances between the COO group of E43 and the two nitrogen atoms, N_ε_ and an N_η_, of R51 being 2.77 Å, but the expected salt bridge did not form spontaneously during the all-atom optimization. Examination of the starting geometry showed that when hydrogenation had been performed by MOPAC, the ionizable hydrogen on E43 had been placed on the O_ε_ nearest to N_ε_, and therefore migration from that atom to the guanidine of R51 was not possible. The origin of this problem was traced to a limitation in the hydrogenation procedure used in MOPAC. When carboxylate groups are hydrogenated an ambiguity exists because both oxygen atoms are in a similar environment. To resolve ambiguities of this type in MOPAC, the hydrogen atom would be added to the oxygen atom that had the longer bond-length to C_δ_. When the hydrogen atom in the starting geometry was moved from the original O_ε_ to the other O_ε_ and the unconstrained geometry optimization re-run, the salt bridge did form spontaneously.

### Incorrect salt bridges precluded

Having established that the model correctly predicted that ionized sites exist, another possible fault would be that the model might incorrectly predict ionization to occur in sites that should in fact be neutral. In proteins, glutamine does not normally ionize, so this possibility was tested by modeling a putative salt bridge consisting of Q69(+) - D82(−) in an otherwise neutral MTH1. In 3ZR0, for this system the smallest N_ε_ - O_δ_ distance was 2.72 Å in chain A and 2.76 Å in chain B, well inside the normal four Å limit for a salt bridge. When only hydrogen atom positions were optimized, the resulting salt bridge system was 4.8 kcal∙mol^−1^ higher in energy than that of the neutral system, and when an unconstrained geometry optimization was performed the difference in energy relative to the equivalent system that did not have the test salt bridge increased to 6.0 kcal∙mol^−1^. In both cases the energetics indicate that Q69 should exist as the neutral residue, and therefore that the correct prediction had been made by the computational model.

### The Nudix box

**Fig. 9:**
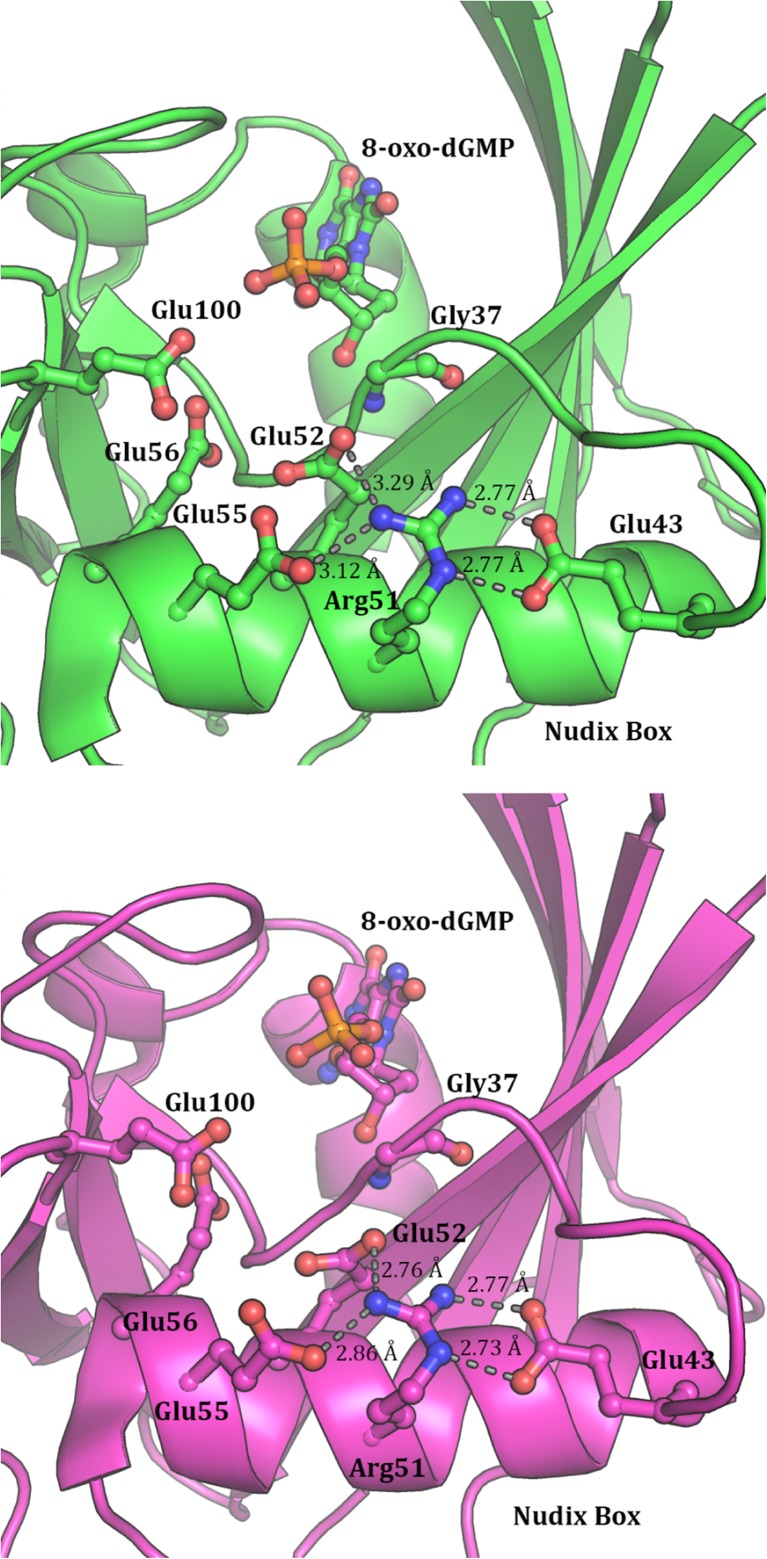
Environment of Arg51 in the Nudix box, PDB (*top*) and PM7 (*bottom*), showing nearby glutamic acid residues

MTH1 contains a catalytic site, the Nudix box, composed of a hairpin fold with one leg being a short α helix and the other a single strand of a β sheet. The characteristic motif of this structure is GX_5_EX_7_REUXEEXGU, where U = I, L or V, and X is any residue. In MTH1, this structure starts at residue 37 and the Nudix box has the sequence G37-X_5_-E43-X_7_-R51-E52-L53-X-E55-E56-X-G58-L59. Nine of the 23 residues in this structure are highly conserved. This set of nine can be further divided into two groups, one consisting of the hydrophobic residues G37, L53, G58, and L59 and the other composed of the potentially ionizable residues E43, R51, E52, E55, and E56. The helix is amphipathic with all the ionizable residues being on the same side of the α helix. Three of the glutamic acid residues (E43, E52, and E55) are near enough to form hydrogen bonds with the R51 and are therefore potential candidates for forming a salt bridge. Two of these, E52 and E55, form monodentate hydrogen bonds with the same N_η_ atom on R51. The third, E43, forms a bidentate side-on interaction with N_ε_ and the other N_η_, as shown in Fig. 9. Bidentate carboxylate argininium interactions have been predicted [31] to be more stable than monodentate, so it is likely that the R51-E43 system forms a salt bridge. This conclusion is reinforced by the small separation of the donor-acceptor atoms, 2.8 Å, which is typical of a carboxylate-argininium salt bridge [32]. Although the other two carboxylic acid groups are in a similar environment, the donor-acceptor distances are significantly larger, averaging 3.2 Å. Several of these negatively-charged residues are potential ligands of the magnesium cofactor of MTH1, i.e., the orientation of the glutamic acid residues resulting from the interaction with the arginine provides an ideal framework for Mg^2+^ binding.

## Discussion

Any discussion of the relationship between an X-ray and PM7 protein structure requires a metric that would allow the similarities and the differences between the structures to be compared. In this analysis, two metrics will be used, the first being a comparison of the overall structure, a global metric, the second being a comparison of local anomalies, mainly relating to interatomic distances and angles.

## Global comparison

### Effect of bias on geometry

When an energy penalty function was added to the calculated ΔH_f_, a bias was introduced that moved the optimized geometry from the unconstrained PM7 minimum toward the unmodified X-ray structure. As the penalty became greater, the structure approached the PDB structure (Fig. 3). This was accompanied by a concomitant increase in strain energy (Fig. 4), representing the increase in ΔH_f_ relative to the unconstrained optimized PM7 structure. With decreasing penalty, only small changes in geometry occurred until a penalty of 2 kcal∙mol^−1^∙Å^−2^ was reached; below that there was a rapid increase in the RMSD between the calculated and X-ray structures. A different pattern took place in the strain, where, as the penalty decreased, the strain energy dropped steadily until at a penalty of 2 kcal∙mol^−1^∙Å^−2^ fully 64 % of the strain energy had been relieved. At that point the strain was 721.6 kcal∙mol^−1^. Below that the strain decreased rapidly to zero.

**Fig. 10:**
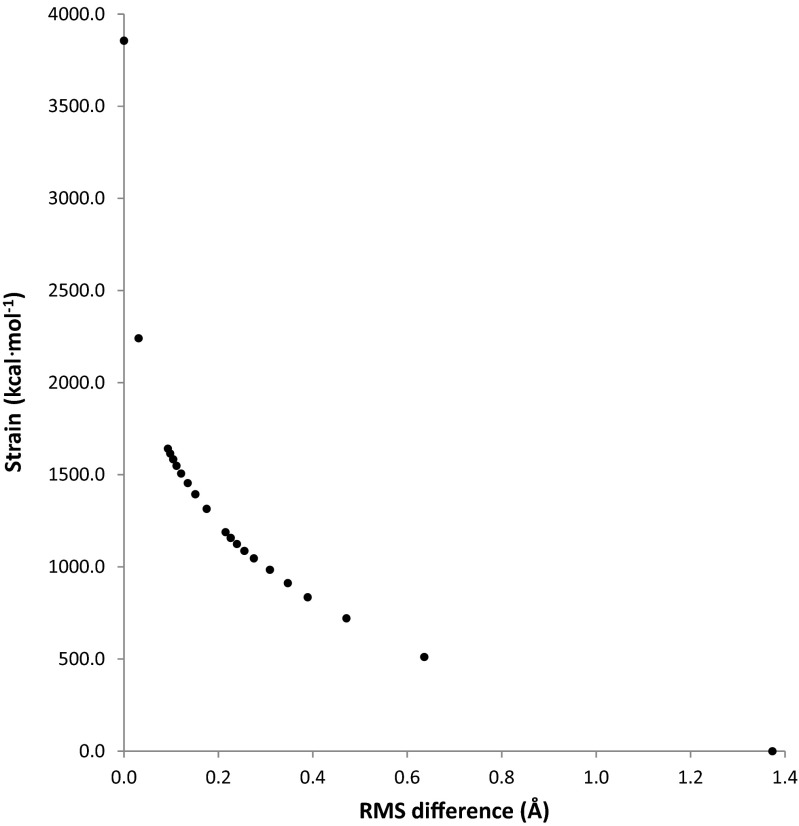
Comparison of changes in heats of formation for various degrees of distortion from PDB geometry for 3ZR0. Un-modified 3ZR0 is represented by the point at RMSD = 0.0. The PM7 geometry is represented by the point at a strain of 0.0 kcal∙mol^−1^

A more useful representation of the effect of geometric errors in the PDB and PM7 structure is a comparison of the change in ΔH_f_ as the RMSD increased (Fig. 10). When the RMSD was zero, the geometry corresponded to the PDB structure, and the strain was at a maximum 3856.3 kcal∙mol^−1^. The first non-zero RMSD calculated was 0.031 Å, at which point the strain had dropped to 2241.2 kcal∙mol^−1^. That is, an average change in each atom’s position of only 0.03 Å resulted in a decrease in strain energy of 42 %. An RMSD of 0.10 Å resulted in a 59 % decrease in strain energy. Such large decreases in strain energy would most likely be due to the correction of errors in interatomic bonding separations in the PDB structure. A list of the largest differences between PDB and PM7 covalent bond lengths is given in Table 3, together with the bond-lengths of equivalent bonds abstracted from the Cambridge Structural Database (CSD) [33]. In most instances, PM7 bond-lengths were nearer than the PDB bond-lengths to the appropriate CSD entry, the exception being P-O, where PM7 predicts the bond-length to be too long by 0.1 Å.

**Table 3:** Largest bond-length differences in Å between X-ray and PM7 geometries of 3ZR0

#		Res.	Atom_1_	Atom_2_	Δ	R_PM7_	R_PDB_	R_CSD_	CSD entry
B	103	Pro	C_β_	C_γ_	−0.249	1.534	1.285	1.522	AGAQOP
B	54	Gln	C_β_	C_γ_	0.212	1.521	1.733	1.525	KATBEN
B	87	Cys	S_γ_	C_β_	−0.191	1.829	1.638	1.802	NALCYS19
B	151	Arg	C_β_	C_γ_	−0.167	1.527	1.360	1.540	UHUCUV
A	61	Val	C_β_	C_γ2_	−0.124	1.523	1.399	1.520	RAKWOQ
B	95	Pro	C_β_	C_γ_	−0.123	1.537	1.414	1.522	AGAQOP
B	1157	8OG	P	O_P3_	−0.121	1.634	1.513	1.523	AEPHOS03
A	1157	8OG	P	O_P3_	−0.109	1.615	1.506	1.523	AEPHOS03
B	1157	8OG	N_9_	C_8_	−0.109	1.435	1.326	1.396	REQMOQ
B	6	Leu	C_γ_	C_δ2_	0.104	1.529	1.633	1.510	XOFWIA
A	1157	8OG	N_9_	C_8_	−0.103	1.442	1.339	1.396	REQMOQ
A	86	Phe	C_γ_	C_δ1_	−0.101	1.395	1.294	1.358	FEPJER
A	3	Ala	C_α_	C_β_	0.098	1.520	1.618	1.515	EZACIR
A	1157	8OG	N_1_	C_2_	0.098	1.361	1.459	1.351	REQMOQ
B	1157	8OG	C_4_	C_5_	0.096	1.417	1.513	1.383	REQMOQ

*CSD*: Cambridge Structural Database [33]. Where multiple bonds are present, the average bond length was used

The assumptions were made that the positions of atoms in the X-ray structure of 3ZR0, resolution 1.8 Å, would be within about 0.2 Å of the actual atomic positions, and that this quantity could be used as an RMSD measure of error in the X-ray structure. At this point (Fig. 10), all larger differences could be attributed to crystal packing and to faults in PM7. Obviously there will be a transitional region where structural errors due to limitations in X-ray analyses and PM7 are both significant, but for the purposes of discussion the assumption can be made that X-ray contributions to error become insignificant at an RMSD of 0.2 Å, this value being significantly larger than any likely error in the PM7 structure. At this point, the resulting strain energy was roughly 1200 kcal∙mol^−1^. All the remaining strain energy can then be assigned to errors [17] in PM7 and to crystal packing energies.

### Crystal packing effects

No direct estimate of crystal packing energies is possible, but an estimate can be made of the effect on the geometry of MTH1 due to the crystal packing. 3ZR0 contains two, presumably identical, molecules of MTH1, so all geometric differences between the two molecules must be due to environmental, i.e., crystal packing, differences. Given that the RMSD in the geometries of the two molecules present in un-modified 3ZR0 was 0.72 Å and that the energy released on forming a crystal must be very small, it follows that some motions in the order of 0.7 Å require very little energy. This extreme flexibility is vividly demonstrated in 3ZR0 where the functional groups of K131-E152 are 5.71 Å apart in MTH1 A and only 2.75 Å apart in MTH1 B. Indeed, the geometric consequences of the crystal packing appear to be so large that the RMSD between the aqueous form of MTH1 and the crystal form might be larger than between that of the two MTH1 molecules in 3ZR0. Using this metric, much of the RMSD (1.37 Å) between the fully optimized aqueous-phase PM7 geometry and the crystal geometry in 3ZR0 could be attributed to the absence of crystal packing in the PM7 system.

An alternative measure of geometry that should be less dependent on crystal packing would be the volume of the system. For this, the volume enclosed by the solvent accessible surface (SAS), calculated using the COSMO technique [23], is suitable. For the PDB starting geometry for MTH1 A+B, this volume was 48550.4 Å [3], and for the PM7 fully optimized geometry 44824.6 Å [3]. Thus the volume of the optimized geometry was 7.7 % smaller than the X-ray geometry. This implies that the dimensions of the optimized geometry were about 2.5 % smaller than that observed. These results confirm that PM7 optimized geometries can reproduce protein volumes with good accuracy and that optimized geometries do not deviate dramatically from observable structures.

### The importance of correct ionization

Because MTH1 is crystallized at a very low pH, resulting in a charge distribution on the surface of the protein that can be expected to deviate from the in vivo situation, it is important that the charge interactions under differing ionization conditions are correctly modeled. When the geometry of an initially almost completely neutral system was optimized and the result compared with the equivalent system in which every ionizable site had been ionized, the RMSD was relatively small. When all potentially ionizable sites were ionized, it resulted in the formation of 13 classic salt bridges, all of which were on the surface of the protein, where they contribute to the stability of the protein [34]. Of the ionized sites that did not participate in salt bridge formation, two were isolated surface residues that were stabilized by solvation, and two were buried sites, D119 and D120. The remaining ionized sites were sulfate dianions and the phosphate anion; these all formed strong hydrogen bonds with nearby protein residues.

The largest differences between the completely neutral and ionized structures in the optimized geometries were caused by the different ionizations within side-chains of residues, and then only in those residues which were very flexible: that is, where energy changes arising from distortions were small, such as on the surface of the enzyme. Such conditions would not exist in either the Nudix box or in the binding site, regions dominated by a large number of strong hydrogen bonds. Even the presence of a significant net charge, −9, on MTH1 A had only a minimal effect on the overall structure. Because of electrostatic repulsion, a large net charge might be expected to cause a dilation of the system and any such change would be reflected in the volume of the system. However, on ionizing all sites the volume of the optimized geometry decreased from that of the neutral system, 23487.44 Å [3], to 23405.56 Å [3], a reduction in volume of 0.18 %. So not only was there not a significant increase in volume due to electrostatic repulsion, the PM7 prediction was that the volume of the ionized system should decrease by a small amount.

Based on these results when modeling enzyme systems the following guidelines should be used:

- Residues that could potentially participate in salt bridge formation should be ionized. If there is any doubt, a calculation of the ionized and un-ionized putative salt bridge should be run. If the calculated ΔH_f_ decreased when the salt bridge was present, then the existence of the putative salt bridge would be confirmed.
- Unless a potentially ionized site is close to a region of interest, i.e., a catalytic site or binding site, the state of ionization is not geometrically relevant as indicated by the small structural changes upon ionization.
- The state of ionization of individual residues in sites of interest should be determined on a case by case basis.

## Local anomalies

### Hydrogen bonds

Protein secondary structures are stabilized by hydrogen bonds, which are weaker than covalent bonds, and can be used as a sensitive test of the ability of a computational method to reproduce the backbone structure. In this analysis, only hydrogen bonds involving the protein or the substrate 8-oxo-dGMP will be discussed; inter-water, water-protein, and related hydrogen bonds will be ignored.

The unsigned median, mean difference, and average signed error between the PDB and PM7 donor-acceptor distances were 0.08 Å, 0.14 Å, and −0.09 Å, respectively. A small number of significant changes accounted for the large increase in going from the unsigned median to mean difference. Deviant hydrogen bonds were:

R51 - Q54: In the PDB starting geometry, the O - H_ε_ distance in chain A was 3.45 Å; on optimization using PM7 this decreased to 2.07 Å. Q54 is on the surface of the protein and its side-chain is extremely flexible, as evidenced by the fact that the orientation of Q54 in chains A and B in the PDB structure are markedly different; in chain A the O(Q54) - N_ε2_ (R51) separation is 4.42 Å, whereas in chain B the equivalent distance is 6.08 Å. The difference in the PDB and PM7 geometries in this region can then be explained by this flexibility, that is, quite small changes in energy were resulting in large changes in the conformation of side chains. This flexibility also explains several of the other, smaller, differences in hydrogen bond lengths.

D119-W123: This hydrogen bond lies at the end of the β-antiparallel sheet just before the start of a hairpin bend to one of the α-helices. At this point in the PDB structure the β-chain had started to separate from the sheet, the donor-acceptor distance being over 3.4 Å in both chains A and B, but in the PM7 structure the equivalent distance was less than 3.0 Å and a strong hydrogen bond was present. This resulted in an error in the hydrogen bond-length of about 0.6 Å, and represented the largest difference in the set of important hydrogen bonds between the PM7 and PDB structures. A similar shortening of a hydrogen bond occurs in Phe133-Val67, where the backbone donor-acceptor distances in the PDB structures for chains A and B were 3.23 Å and 3.05 Å, respectively; using PM7 these separations decreased to 2.72 Å and 2.71 Å, the donor-acceptor distances decreasing by 0.51 Å and 0.34 Å. Changes in hydrogen-bond lengths of this type are different in nature from those where flexibility is involved. In the current cases the shortening (strengthening) of the hydrogen bond is likely caused by a fault in PM7. Thus in the F133-V67 case, PM7 predicted an unusually close contact, 4.18 Å, between one of the C_ε_ of the phenyl ring and a C_γ_ of the valine; in the PDB structure this distance is 4.74 Å. This effect is typical of a known fault [17] in PM7 that causes non-interacting groups to approach closer than expected when a nearby strong non-covalent interaction, here a hydrogen bond, is present.

All hydrogen bonds within the α-helices and the β-antiparallel sheet were reproduced with good accuracy. The dimensions of the chain sections that participated in the β-antiparallel sheet were reproduced within 3.5 %. In the larger α-helix, the length of the helix, as predicted by PM7, was 14.8 Å, about 2.4 % shorter than the 15.2 Å reported in the PDB structure.

What was not reproduced well by PM7 was the inter-chain separation in the β-antiparallel sheet, which was under-estimated by 8.0 %. Using MolProbity [24, 25], both the original PDB structure and the fully optimized PM7 structure for 3ZR0 were examined. MolProbity is a protein structure validation program that can be used for identifying unusual or unexpected systems that might be indicative of artifacts in the structure. In an initial run using MolProbity, the unmodified geometry resulting from a MOPAC calculation was used, but the resulting clashscores were very large. Clashscores are a measure of the incidence and magnitude of unrealistically close non-covalent interactions. The origin of the large clashscore was traced to the presence in the MOPAC geometry of hydrogen atoms on water molecules, a prerequisite for a realistic computational chemistry model; this can be contrasted with the default in MolProbity of not including hydrogen atoms on water molecules, presumably because MolProbity was designed for examining X-ray structures. By deleting the hydrogen atoms from MOPAC calculations and instead using the MolProbity hydrogenation, results were obtained that could be compared with those of crystallographic analyses. A summary of the results for various calculations is shown in Table 4. Comparing the differences showed a large increase in the clashscore in going from the PDB to the PM7 structure. In the PDB structure, the clashscore was 5.63, while the equivalent value in the PM7 structure was 17.88. This fault was traced to a severe underestimation of the hydrogen-bond length for the longer, thus weaker, bonds. In the extreme case mentioned earlier this amounted to 0.6 Å. Shorter hydrogen bonds were reproduced with improved accuracy, being typically about 0.1 Å too short. This error in the longer hydrogen bond lengths was the main cause of the overall decrease by 7.7 % in the predicted volume of the protein and likely contributed significantly to the concomitant RMSD error in the backbone of 0.38 Å.

**Table 4:** Summary of MolProbity results for X-ray and PM7 structures

Metric	X-ray		PM7		PM7 with penalty of 1.0 kcal∙mol^−1^∙Å^−2^		PM7 + H-H repulsion	
Clashscore, all atoms	5.63	92nd percentile	17.88	38th percentile	2.21	99th percentile	9.44	74th percentile
Poor rotamers	10	3.76 %	12	4.51 %	10	3.76 %	11	4.14 %
Ramachandran outliers	0	0.00 %	2	0.67 %	0	0.00 %	0	0.00 %
Ramachandran favored	296	99.00 %	278	92.98 %	292	97.66 %	285	95.32
MolProbity score	1.74		2.70		1.51		2.29	
Cβ deviations > 0.25 Å	2	0.73 %	0	0.00 %	0	0.00 %	0	0.00 %
Bad backbone bonds	8/2558	0.31 %	19/2558	0.70 %	12/2558	0.47 %	18/2558	0.70 %
Bad backbone angles	4/3458	0.12 %	16/3458	0.52 %	14/3458	0.40 %	25/3458	0.72 %
Bond length outliers	8/310	2.5 %	13/310	4.2 %	7/310		12/310	3.8 %
Bond angle outliers	3/310	1.0 %	16/310	5.5 %	14/310		23/310	7.4 %

Problems involving PM6 and PM7 predicting unexpectedly close contacts had already been reported. Řezáč and Hobza found [14] that PM6 strongly underestimated hydrogen-hydrogen intermolecular distances in hydrocarbons. They were able to correct this error by adding a repulsive term to all pairs of hydrogen atom interactions; this modification was incorporated into the method PM6-D3H4 [14]. A similar fault was also found [17] in PM7 where sections of a protein chain became unrealistically close together. When the repulsive term in PM6-D3H4 was added to PM7 and the geometry re-optimized, the clashscore decreased from 17.9 to 9.4. Examination of the remaining clashscores revealed that, although the addition of the specific H-H repulsion term to PM7 resulted in the elimination of unrealistically close contacts involving pairs of hydrogen atoms, other unrealistic close contacts were still present. Of these, the most important involved carbon-carbon, carbon-oxygen, and carbon-hydrogen close contacts. These errors involve very weak interactions, so it is likely that they, too, could be eliminated by the addition of similar repulsion terms.

Another measure of accuracy for protein structures can be generated by a comparison of various interatomic distances and angles with standard values, and for this task MolProbity was also ideally suited. Of the 13 bond-length outliers MolProbity reported for PM7, six involved C_ε1_ to either N_ε2_ or N_δ1_ in a histidine where PM7 predicted the C-N bond to be ∼1.37 Å, significantly longer than the typical bond-length of ∼1.33 Å. Four of the remaining outliers involved an unusually long, 1.55 Å, S-O bond in a sulfate dianion; in 3ZR0 such bonds have a length of 1.46 Å. In three of these the unusually long bond involved a very strong salt bridge to the ionized N_ε_ of a lysine, while the fourth outlier involved a multiply hydrogen-bonded oxygen on the sulfate. These environmental effects would tend to increase the S-O bond length. Conversely, a comparison [4] of the geometries of various sulfate groups predicted by PM7 with reference geometries reported in the CSD, showed that the S-O distances predicted by PM7 were systematically too long by about 0.04 Å, or 2.7 %. This suggests that the differences in the S-O bond length must be attributed to a fault in PM7. Of the remaining three outliers, one involved an anomalously long N_ε_ - C_ζ_ bond in one of the 15 argininium groups, R51, a residue near the middle of the Nudix box. PM7 predicts this bond-length to be ∼1.39 Å, considerably longer than the reported ∼1.33 Å. On average, PM7 predicts the N_ε_ - C_ζ_ bond-length to be about 1.36 Å, i.e., about 0.03 Å too long. The unusual environment in R51 (the guanidinium ion forms four strong hydrogen bonds with three nearby carboxylate groups) resulted in an additional elongation of 0.03 Å. This was sufficient for MolProbity to flag it as an outlier. Another outlier was the C-N backbone bond in one of the 32 glycine residues, where PM7 predicted the peptide bond between Gly36 and Gly37 to be 1.39 Å long, versus the PM7 average predicted length of 1.36 Å. In general, PM7 predicts the C-N peptide bond to be too long by 0.03 Å, and this, together with the unusually long bond between Gly36 and Gly37, almost certainly the result of environmental conditions, caused it to be flagged as an outlier. The final outlier occurred in one of the Asp residues, where the optimized PM7 structure predicted that Asp120 should be neutral. This resulted in a significant difference in C-O bond-lengths, 1.21 and 1.34 Å, resulting in the longer of these being flagged by MolProbity as an outlier. MolProbity automatically assigns a charge of −1 to carboxylate groups, although in this particular case Asp119 is adjacent to Asp120, and there is some debate in the literature about whether one or both of these Asp residues is charged. If, in fact, Asp120 is not charged, the bond lengths predicted by PM7 may be closer to those in the enzyme system in vivo than the average carboxylate bond lengths.

MolProbity reports 18 bond-angles in the PM7 geometry that were more than four standard deviations from the mean. A representative example is provided by phenylalanine, where MolProbity flagged the C_α_-C_β_-C_γ_ angle in 11 of 22 phenylalanine residues as being outliers. PM7 predicted the average value of this angle to be 110.9° versus the average in the PDB structure of 113.6°. Thus the conclusion can be made that PM7 systematically underestimates this angle by 2.7°. A similar interpretation can be made for the other angles flagged by MolProbity; for any given type of angle that was flagged, typically half were within the assigned limits and half were outside. In every case where there were two or more outliers involving the same type of angle, the predicted angles were either all larger than or all smaller than the target values, implying that the outliers were due to a fault in the computational method and not due to local environmental conditions.

MolProbity was developed as a tool for validating X-ray crystallographic structures of proteins and nucleic acids. As such, it was not intended or designed for the validation of calculated structures. X-ray structures are fundamentally experimental in nature, so anomalies reported by MolProbity reflect deviations from reference experimental values. These anomalies could, of course, be generated by features of the specific system, e.g., structural or positional disorder, or by technical issues relating to the X-ray analysis. Anomalies reported by MolProbity in structures predicted using computational methods, on the other hand, would be caused by two very different reasons.

First, errors in the computational method could produce a systematic fault. Examples of these, as noted earlier, would be the 2.7 % increase in the S-O bond length in the sulfate dianion, the 2.7° error in the C_α_-C_β_-C_γ_ angle in phenylalanine and the underestimated repulsion between hydrogen atoms. All these faults could be removed by making appropriate modifications to the method.

The other type of anomaly in calculated structures would be caused by local environmental conditions. A dramatic example is R51, where the underlying systematic error in PM7 in the N_ε_-C_ζ_ bond-length of 0.03 Å in argininium was exacerbated by the unusually strong hydrogen bonding environment of the guanidinium, thus resulting in an overall error of 0.06 Å or 4.5 %. Of the 15 arginine residues in 3ZR0, only R51 in chain B was flagged by MolProbity as having an outlier bond length. Another example can be seen in the peptide bond between G36 and G37 in chain A, where, as a result of local environmental conditions, possibly related to the role of G37 in the Nudix box, the C-N backbone was elongated by an additional 0.03 Å to 1.38 Å, 0.06 Å longer than the normal peptide bond length. Of the 302 peptide bonds, this was the only one reported by MolProbity.

Environmental conditions are real in the sense that they are a structural feature of proteins, and in particular enzymes, biochemical macromolecules ideally suited for catalyzing reactions. Therefore the fact that they can perturb individual bond lengths or angles should not be regarded as an error (so long as it is demonstrated not to be a systematic error) in the computational model. Rather, they should be regarded as a feature of the system being modeled.

### Nudix box

MTH1 is a nucleotide diphosphate kinase that preferentially hydrolyzes 8-oxo-dGTP to give pyrophosphate and 8-oxo-dGMP. Operations of this type are catalyzed using the Nudix box (the conserved 23 residue structure), and a cofactor. In MTH1, this cofactor would be a Mg^2+^ complex. In 3ZR0, only the product of hydrolysis, 8-oxo-dGMP, is present; the Mg^2+^ and the pyrophosphate group are both absent.

There are four glutamic acid residues (E43, E52, E55, E56) in the Nudix box, with, in MTH1, a fifth residue, E100, in close proximity. E43 is the most distant from the active site and forms a strong face-on salt bridge with N_ε_ and an N_η_ of R51. On the other side of R51 are the carboxylate groups of residues E52 and E55. These each form a single hydrogen bond to the other N_η_ of R51. In this structure R51 appears to behave as a positioning mechanism; the salt bridge with E43 effectively eliminates it as a candidate for salt bridge formation with other residues, but the electrostatic field of its guanidinium ion helps hold in place the carboxylate groups of E52 and E55.

The environment of E56 in chain B is unusual in that it forms a strong and stable pseudo salt bridge with K23. A water molecule, HOH2011, forms strong hydrogen bonds with both K23 and E56. This water appears to act as a charge-relay in that, although the electrostatic field of lysinium can interact with the carboxylate of E56, its influence is moderated by the intervening water molecule. This results in the carboxylate being held in place as if in a salt bridge, but with its electrostatic effect still available for binding to the helper magnesium complex. Whether this is an artifact of PM7 or is a real phenomenon is unclear.

In summary, a comparison of the catalytic site environments (Fig. 9) shows that the essential features of this structure are preserved by PM7.

### Substrate binding site

Examination of the X-ray structure of 3ZR0 shows that the binding site of 8-oxo-dGMP in MTH1 involves five hydrogen bonds between the substrate guanine group and the enzyme residues N33, D119, and D120. Of particular interest is the D119 interaction in which the O_δ_-O_6_ distance is unusually short, 2.43 Å in chain A (Fig. 2) and 2.30 Å in chain B. Such short distances are unusual in hydrogen bonds [20], and occur only when a charged species is involved. An example is the sodium hydrogen acetate CSD [33] entry NAACET06, where the equivalent O-O distance in the [CH_3_COO-H-OOC-CH_3_]^−1^ system is 2.47 Å. This strongly implies that the D119-guanine system has a net negative charge.

In principle, guanine can exist in two forms: a keto form where N_1_ is protonated, and an enol form where O_6_ is protonated. Svensson et al. [19], referring to the bound 8-oxo-dGMP in MTH1 stated, “We speculate that the 1- and 6-position is critical for the specificity and that the 6-enol-8-keto form of 8-oxo-dGTP is able to donate and accept hydrogen bonds at these specific positions.” That is, if 8-oxo-dGMP in MTH1 were to exist in the enol form then D119 would be ionized and the resulting carboxylate would form a strong hydrogen bond with the hydrogen on O_6_. Conversely, a high-level calculation reports [35] that “(the) enol tautomer … is not stable in the aqueous phase. It is 8.7 kcal∙mol^−1^ higher in free energy than (the keto form) leading to a population in the aqueous phase of 4 ∙10^−7^.” Due to the increase in energy in going from keto to enol, these results suggest that almost all guanine in solution, and presumably in the docking site of MTH1 (unless modified by the docking site), would be in the keto form.

**Fig. 11:**
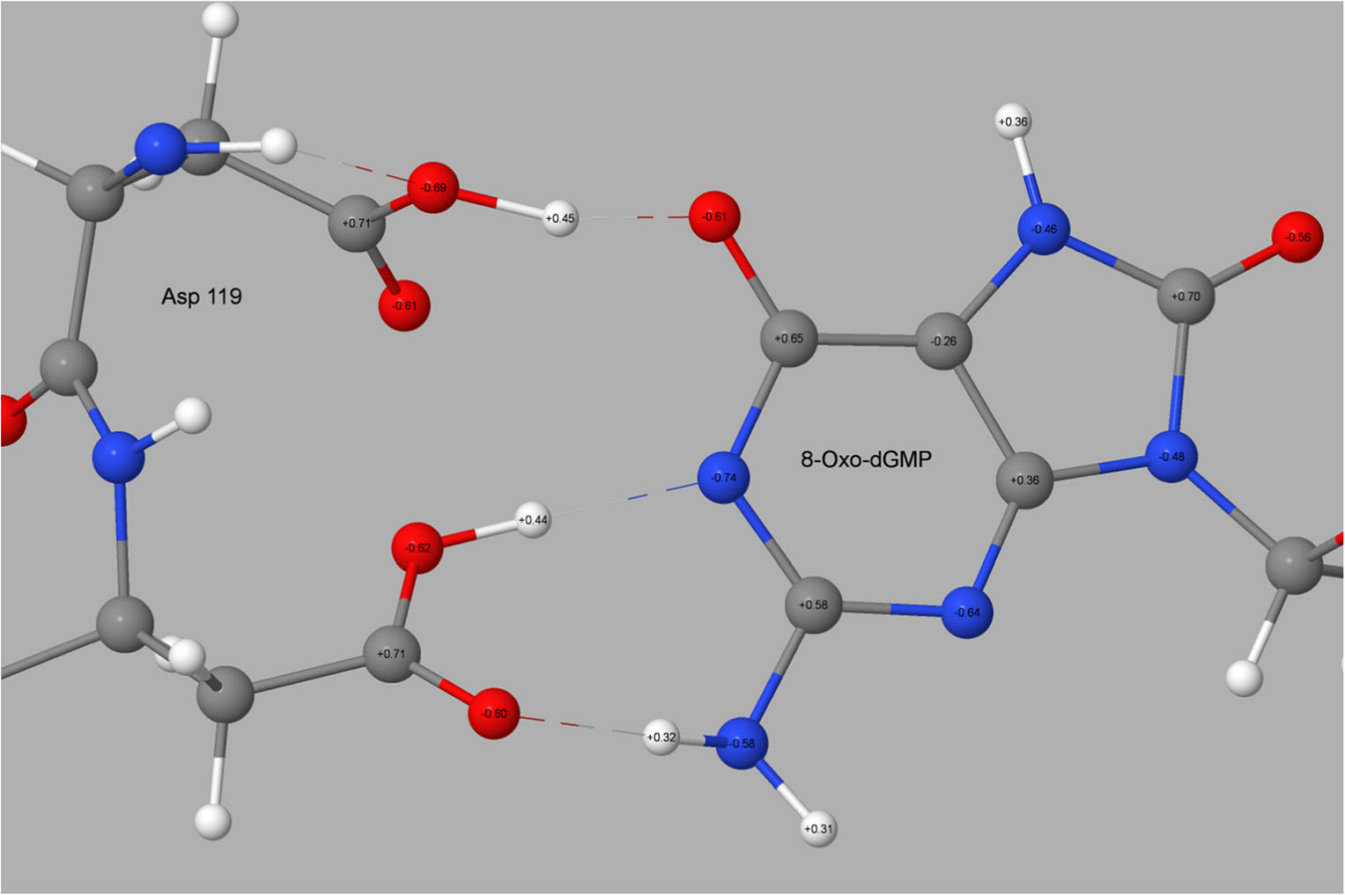
PM7 atomic partial charges for Asp119, Asp120, and 8-oxo-guanine ring of 8-oxo-dGMP. Net charge on the 8-oxo-guanine ring: −1.05

In the enol starting geometry, the 8-oxo-guanine group was in the 8-keto-6-enol tautomeric form and neutrally charged, and Asp119 was ionized, this being the structure presumed to exist in the docking site. However, after an unconstrained full optimization was performed, the proton on O_6_ in 8-oxo-dGMP in both A and B chains was found to have migrated to form a covalent bond with the D119. This implied that the assumed initial configuration had been incorrect, and that the binding site and aqueous environments were significantly different. An examination of the charge distribution in 8-oxo-dGMP after geometry optimization revealed (Fig. 11) that the guanine group had a net charge of -1.05. This suggests that, in MTH1, 8-oxo-dGMP exists as the dianion, with one negative charge on the phosphate group, a neutral deoxyribose, and a negative charge on the guanine. Now an explanation for the short O_δ_-O_6_ distance can be made: instead of the proposed [19] hydrogen bond existing between the carboxylate and hydroxyl (as would be the case if 8-oxo-dGMP were in the enol form), the results of this calculation imply that it exists between the O_6_ on the guanine (keto form) anion and the carboxylic acid.

This description could be extended to explain the D120-guanine interaction. Earlier, the assumption had been made that all ionizable sites would be ionized, but that, in the vicinity of the D119-D120 guanine complex, the presence of a negative charge on either D119 or the guanine would alter the pK_a_ of D120, with the result that D120 would likely be neutral, and therefore would form a hydrogen bond with N_1_, and the hydrogen atoms on N_2_ of guanine would form a hydrogen bond with an O_δ_ of D120. After D120 was neutralized and chain A optimized once more, a comparison of the environment of the residues D119, D120, and guanine showed an improved fit with the X-ray structure. The hydrogen bonding pattern that was expected based on the X-ray structure was obtained, the calculated O_δ_-O_6_ distances, at 2.49 Å and 2.51 Å, being similar to those in 3ZR0, 2.43 Å and 2.30 Å. If the hydrogen atom on D119 were allocated to the guanine group, then the charge on guanine would be −0.60, but if it were assigned to D119, the charge on the guanine would be −1.05.

This analysis gives rise to a dilemma. It appears to imply that the guanine in 3ZR0 would exist either in the enol form or in the anionic keto form, depending on how it is viewed. Obviously a very short and very strong hydrogen bond exists between O_δ_ on D119 and O_6_ on the guanine. This makes the assignment of the proton to one or other group difficult. If the proton were to be assigned to the guanine O_6_, then guanine could be regarded as neutral and in the enol form. On the other hand, if the proton was assigned to D119 then the guanine would be in the keto form and would have a unit negative charge. Both descriptions would result in a strong hydrogen bond existing between the keto oxygen on guanine and the -COO group on D119. That is, the arbitrary choice of which atoms in the hydrogen bond are the donor and acceptor determines whether the guanine exists in the anionic keto or enol form. Tautomers normally exist as well-defined species with an energy barrier between them. In this case, the barrier is either very small or non-existent, and the use of the terms keto and enol as referring to discrete entities would seem to be inappropriate.

In an attempt to clarify the description of the binding site, geometry optimizations of a solvated acetate-8-oxo-dGMP complex were performed using PM7 and, in a separate calculation, the B3LYP [36] DFT method within Gaussian09 [37]. Both optimizations gave similar results. During the optimizations, the hydrogen atom, initially on O_6_, migrated to the acetate and resulted in the O_δ_-H and O_6_-H distances being 1.09 Å and 1.45 Å, respectively, for PM7, and 1.09 Å and 1.39 Å, respectively, for B3LYP. These results implied that a carboxylic acid group and not a carboxylate was present: that D119 was not ionized, and that the guanine exists as the anion.

### Role of the PDB file

A prerequisite for this work was that a starting geometry, the PDB file 3ZR0, existed. Small errors in relative atomic positions, with the largest being about 0.25 Å, resulted in very large errors in calculated heats of formation. These small geometric errors can now be easily corrected by computational chemistry modeling. In turn, an unconstrained geometry optimization starting with the PDB structure resulted in an RMSD between the PM7 predicted structure and the X-ray structure of about 1.37 Å. Two causes for this large difference can be identified. First, methods such as PM7 focus on energies which are dominated by covalent interactions, and semiempirical methods for modeling non-covalent interactions were, until recently, of low accuracy. With the advent of dispersion and post-hoc hydrogen bond corrections, these interactions can now be modeled with increased accuracy. This results in a more realistic representation of hydrogen bonding in α-helices and β-sheets, that is, in the secondary structure. Even with this improvement, it is possible that much of the error in the tertiary structure can be attributed to errors in modeling very low energy interactions [17].

A second source of potential error is the neglect of crystal packing. The model used in the simulations described here is the protein system in aqueous phase, not in a crystal environment. Currently, simulation of the crystal structure of MTH1 using PM7 is impractical because of the absence of a complete crystal structure. Many water molecules and other entrained species were not reported in the X-ray analysis. The absence of a complete crystal structure precludes any meaningful modeling of proteins in the crystalline phase, although a simulation of a much smaller system, deamino oxytocin heptahydrate, a decapeptide, did show good agreement between the PM7 and X-ray structures (CSD entry DUPFAV), with the predicted unit cell geometry being (a = 22.96 Å, b = 9.15 Å, c = 27.17 Å, α = 90.33°, β = 102.08°, γ = 89.49°) versus the reported (a = 23.04 Å, b = 9.04 Å, c = 27.27 Å, α = 90.00°, β = 102.24°, γ = 90.00°). It is a well-accepted fact that crystal packing can result in distortions to the system, and this is evident from the RMSD of 0.72 Å when the two MTH1 proteins in 3ZR0 are compared.

### Computational effort

All calculations were performed using a MacPro workstation equipped with 2x2.93GHz 6-Core Intel Xeon processors and 16Gb of RAM. With the exception of the fully unconstrained and mildly-constrained geometry optimizations on the 305 residue 3ZR0, which took about 6 CPU days, all calculations took about one CPU day. With 16Gb of RAM, 12 calculations could be run simultaneously.

## Conclusions

Modeling of enzyme systems involving about 150 residues using semiempirical methods, in this example PM7, can provide a chemically useful description of the various structures involved. Given as a starting point an X-ray structure from the Protein Data Bank, preconditioning—mainly hydrogenation—to create a starting model is straightforward. Once a starting model is available, the transition from the X-ray structure to a simulated structure suitable for further computational analysis can then be performed. Geometric features, such as the binding site and the catalytic site, (here, the Nudix box in the protein MTH1) are reproduced with high accuracy. In contrast to X-ray structures, which are a static description of experimental observations, computational methods now allow modeling of hypothetical chemical operations, such as mutating residues and the investigation of catalytic mechanisms.

Although the effect of altering the ionization state of residue side chains has little effect of the overall structure, correct assignment of charged sites can significantly improve modeling of enzymatic structures determined from X-ray crystallography. Crystallization of biological enzymes often requires gross manipulation of ionic strength and pH, so determining the correct ionization of proteins when modeling physiological conditions can greatly improve the understanding of molecular interactions, especially the type that occur in binding and catalytic sites.

Simulation of an entire enzyme system using a single method has a few limitations. Because of the very large computational cost, molecular dynamics simulations using quantum chemistry methods are still impractical. Small distortions of the same magnitude as the distortions resulting from crystal packing occur. When PM7 is used, some non-covalent contact distances will be shorter than expected. Fortunately, this specific error only affects regions of a protein where very weak non-covalent interactions are present, and would therefore have little effect on the modeling of active sites, regions where non-covalent interactions such as hydrogen bonds are usually quite strong.

There are distinct advantages in using a single method. All well-understood phenomena—ΔH_f_, hydrogen bonds, electrostatics, structural motifs, such as helices, sheets, hairpin bends, etc.—are reproduced with useful accuracy. More important, phenomena that are less well understood, such as those that occur in the active site, can be modeled with the same degree of confidence. When a single method is used there are obviously no issues of the type that occur when multiple methods are used; for example, there are no interface issues to contend with.

The case has been made that using semiempirical methods for modeling proteins and the phenomena that occur in them is both reliable and practical. An examination of the structure of 8-oxo-dGMP bound in MTH1 leads to the conclusion that discussion of whether the guanine group exists as a discrete keto or enol form is moot, because the absence of a significant barrier between the two tautomers in the in situ structure precludes their discrete existence.

Two types of errors were identified in the PM7 semiempirical method. One affects some specific bond lengths and angles. Errors of this type are of the order of 0.04 Å and 2 to 3 degrees, but do not appear to affect the computational simulation significantly—meaning, few bonds or angles are distorted enough to be flagged as outliers, and the small differences should not be significant enough to affect the chemistry. The other type of error involves an under-estimation of steric repulsion energies that resulted in large MolProbity clashscores. The largest of these errors involved H-H interactions. Using a proposed post-SCF correction [14] the clashscore was reduced by about 50 %. If other post-SCF corrections involving C-C, C-O, and C-H were made, it is likely that the remaining clashscore could be eliminated.
